# Quercetin Improving Lipid Metabolism by Regulating Lipid Metabolism Pathway of Ileum Mucosa in Broilers

**DOI:** 10.1155/2020/8686248

**Published:** 2020-09-18

**Authors:** Mi Wang, Yanjun Mao, Bo Wang, Shanshan Wang, Han Lu, Linlin Ying, Yao Li

**Affiliations:** Institute of Animal Nutrition, Northeast Agricultural University, Harbin, Heilongjiang, China

## Abstract

This study is aimed at evaluating the regulatory mechanism of quercetin on lipid metabolism in the ileum of broilers to better understand these pathways decreasing abdominal fat. 480 chickens were randomly divided into 4 groups (control, 0.02% quercetin, 0.04% quercetin, and 0.06% quercetin). Breast muscle, thigh muscle, and abdominal fat pad were removed and weighed at 42 d of age. Serum was obtained by centrifuging blood samples from the jugular vein (10 ml) to determine high-density lipoprotein (HDL), total cholesterol (TC), low-density lipoprotein (LDL), triglyceride (TG), leptin, and adiponectin using ELISA. About 5 g of the ileum was harvested and immediately frozen in liquid nitrogen for RNA-seq. Then, the confirmation of RNA-seq results by the Real-Time Quantitative PCR (RT-qPCR) method was evaluated using Pearson's correlation. Compared with control, abdominal fat percentage was significantly decreased with increasing quercetin supplementation, and the best result was obtained at 0.06% dietary quercetin supplementation (*P* < 0.01). Breast muscle percentage was significantly decreased at 0.02% quercetin (*P* < 0.01), and thigh muscle percentage tended to increase (*P* = 0.078). Meanwhile, 0.04% and 0.06% quercetin significantly decreased TG (*P* < 0.01), TC (*P* < 0.01), and LDL content (*P* < 0.05) in serum. Serum leptin and adiponectin contents were significantly increased by 0.04% and 0.06% dietary quercetin supplementation, compared with the control (*P* < 0.01). Analyses of Gene Ontology (GO) and Kyoto Encyclopedia of Genes and Genomes (KEGG) database were used to identify differently expressed genes and lipid metabolism pathways. Quercetin decreased abdominal fat percentage through regulating fat digestion and absorption, glycerophospholipid metabolism, AMPK signaling pathway, fatty acid degradation, and cholesterol metabolism.

## 1. Introduction

Meat is an important protein source, and chicken is one of the most popular food commodities in the world and the second most preferred meat for Chinese consumers. The fat of meat is an important component in meat quality and impacts animal productivity. The breeding of poultry focused on increasing growth performance and improving breast and thigh meat yields over the past decades. Growth performance made great progress; however, there has been excessive deposition of abdominal fat. In order to reduce fat deposition and improve feed efficiency, different feed additives have been adopted in broiler production. As a kind of safe feed additives, quercetin is impressive [1].

Quercetin is a flavonoid abundant in onions, apples, tea, and red wine, which exhibits antiviral, antitumorigenic, and anti-inflammatory effects [2–5]. Moreover, chronic administration of quercetin markedly improved dyslipidemia, hypertension, and hyperinsulinemia; reduced body weight gain; and increased blood adiponectin levels in obese Zucker rats [6]. Quercetin reduced hepatic fat accumulation in mice fed a high-fat diet and high-sucrose diet [5, 7]. It also decreased TG accumulation in cultured adipocytes in vitro [8]. Significant decreases in serum TG, TC, and epididymal adipose tissue were observed in mice fed a diet supplemented with 0.025% quercetin for 9 weeks [5]. Another study found a significant reduction of visceral fat (-15.5%) in mice fed a diet supplemented with 0.05% quercetin for 20 weeks [7]. Quercetin attenuated abdominal obesity (-37%) in rats fed a high-fat diet supplemented with 0.08% quercetin for 8 weeks [9]. It also exerted protective effects against the development of nonalcoholic fatty liver disease (NAFLD), partly by overexpression of adiponectin and reduction of inflammatory cytokine levels in ob/ob mice [10]. Additionally, it significantly decreased TC contents of the liver, heart, kidney, and small intestine in rats [11].

The intestine plays a vital role in fat digestion and absorption; both the jejunum and ileum were involved in the absorption of fatty acid (FA) in laying hens and broilers [12, 13]. Estimations for endogenous losses of protein and amino acids in the ileum have been published [14]; however, studies on endogenous fat losses in poultry are scant [15]. No examination was done in the specific intestinal segments where the mechanism of quercetin regulated lipid metabolism, and no systematic studies have been reported on quercetin regulating lipid metabolism in the ileum of broilers at the molecular level.

The transcriptome is a necessary link between genomic/genetic information and the biological functions of the proteome. Of these, RNA-seq has been widely utilized to detect differentially expressed genes (DEGs) between two gene expression patterns and causative variants. Many studies of RNA-seq have been conducted in the intestinal mucosa [16], heart [17], uterine [18], and ovarian tissues in broilers [19].

The objective of this study was to evaluate the regulatory mechanism of quercetin on carcass characteristics in broilers. The results of this experiment will provide scientific basis for quercetin application in animal production.

## 2. Materials and Methods

### 2.1. Animal Feeding and Diets

Arbor Acre broilers (480 chickens, 1 day old, healthy, similar body weight) from a commercial company (Yinong Poultry Limited Company, Harbin, China) were purchased and randomly divided into 4 groups with 6 replicates per group and 20 broilers per replicate. The broilers were housed in wire cages (four-stacked cages; width: 52.6 cm; depth: 42.3 cm; height: 38.1 cm). Lighting (16 h light : 8 h darkness) was provided during the 6-week experimental period, and temperature was maintained at 32 to 34°C in the starting 3 days and decreased by 2 to 3°C per week to a final temperature of 24°C. Humidity of the experimental room varied from 60% to 65%. Experimental diets and water were available ad libitum. The composition of the basal diets is shown in Table 1 (NY/T33-2004), with 4 levels of quercetin: 0.00%, 0.02%, 0.04%, and 0.06% of quercetin in the diet. Quercetin dihydrate powder with 97% purity was purchased from Sigma-Aldrich Company and mixed with basal diets and was offered in mash form (5 mm) after grinding according to the methods of Yang et al. in 2020 [20].

### 2.2. Sample Preparation

At the end of experiment (42 d), two broilers per replicate were selected (*n* = 12 per group). After 12 h fasting, blood samples from the jugular vein (10 ml) were obtained from each group and kept in ice. Serum was obtained after centrifugation at 4°C, 3000 × *g* for 15 min, and stored at -20°C until analysis. Breast muscle, thigh muscle, and abdominal fat pad (including fat surrounding the gizzard, bursa of Fabricius, cloaca, and adjacent muscles) from one bird of average BW per replicate were removed and weighed at 42 d of age. To compensate for the differences in carcass weight, these values were expressed as a percentage of carcass weight. About 5 g of ileal mucosa was harvested and immediately frozen in liquid nitrogen, and kept at -80°C for further RNA isolation. All procedures used in this study were approved by the Animal Care and Use Committee of the University. Housing, management, and care of the birds confirmed to the guidelines of the Agricultural Animal in Agricultural Research and Teaching of Heilongjiang Province (HEI Animal Management Certificate No. 11928).

### 2.3. Enzyme-Linked Immunosorbent Assay

Content of high-density lipoprotein (HDL), LDL, TG, TC, leptin, and adiponectin were determined using an enzyme-labeled instrument according to ELISA kit instructions (Nanjing Jiancheng Bioengineering Institute, Nanjing, Jiangsu, China).

### 2.4. RNA Sequencing

In this endeavor, twelve samples of ileal mucosa were sequenced using the HiSeq 2000 System (Illumina, Inc., USA) by Genesis (Beijing) Co. Ltd. The data analysis included sequencing data filtering, read mapping, transcript and gene identification, analysis of differential gene expression, and functional annotation.

### 2.5. Real-Time Quantitative PCR

To confirm differential expression of genes, real-time quantitative polymerase chain reaction (RT-qPCR) assays for 12 randomly selected DEGs in the same RNA samples were determined using RNA-seq. All gene expression levels were measured using RT-qPCR (7500 Real-Time PCR System, Singapore). The primer sequences for broilers (Table 2) were designed to span an intron to avoid genomic DNA contamination using Primer 5.0. Total RNA was isolated from ileal mucosa using TRIzol (Invitrogen, San Diego, CA, USA), and total cDNA was synthesized following the manufacturer's instructions. Briefly, a total 20 *μ*l of reaction mixture containing 5 *μ*l of total RNA, 4 *μ*l of 5 × Prime Script Buffer, 1 *μ*l of PrimeScript RT Enzyme Mix I, 1 *μ*l of Oligo(dT) Primer (50 *μ*mol/l), 1 *μ*l of Random Primers 6 mers (10 *μ*M moI/l), and 8 *μ*l of RNase Free dH_2_O (TaKaRa Biotechnology, Co., Ltd. Dalian, P. R. China) was incubated in the following conditions: reverse transcription at 37°C for 15 min and inactivation of reverse transcriptase at 85°C for 5 min, until temperature was decreased to 4°C. Successful cDNA synthesis was confirmed by amplifying the *β*-actin amplicon using PCR. cDNA was amplified using a 20 *μ*l PCR reaction system containing 2 *μ*l of cDNA, 10 *μ*l of 2 × SYBR Green PCR Mix, 0.4 *μ*l of 50 × ROX Reference Dye II (TaKaRa Biotechnology, Co., Ltd. Dalian, P. R. China), 0.8 *μ*l of PCR Forward Primer, 0.8 *μ*l of PCR Reverse Primer (Sangon Biological Engineering Technology & Service Co., Ltd. Shanghai, P. R. China), and 6 *μ*l of ddH_2_O. The following PCR conditions were used: initial denaturation at 95°C for 30 s, followed by PCR reaction at 40 cycles of 95°C for 5 s and 60°C for 34 s, and melting curve analysis at 95°C for 15 s, 60°C for 2 s, and 95°C for 15 s. The PCR products were verified by electrophoresis on 1% agarose gel and DNA sequencing. Standard curves were generated using pooled cDNA from the assayed samples, and the comparative cycle threshold method (2^−ΔΔCT^) was used for quantifying mRNA levels.

### 2.6. Statistical Analysis

The data from this experiment were subjected to one-way ANOVA as a completely randomized design with 4 treatments and 6 replicates in each treatment. The data were submitted to ANOVA, using SPSS 20.0 software (2011, IBM). Calculated△Ct (corrected sample) = the mean value of the target gene − the mean value of the internal reference gene; △△Ct = △Ct − the mean value of the control group. Differences among treatment means with a probability level of *P*<0.05 were accepted as statistically significant, and all the results were expressed as the “mean values ± standard deviation.”

## 3. Results

### 3.1. Carcass Characteristics

Thigh muscle percentage tended to increase (*P* = 0.078); however, abdominal fat percentage was significantly decreased with increasing quercetin (*P* < 0.01) (Table 3), and breast muscle percentage was significantly decreased by 0.02% quercetin (*P* < 0.01) compared with the control.

### 3.2. Serum Biochemical Parameters

Comparing with the control, quercetin did not affect HDL content (*P* > 0.05); however, 0.04% and 0.06% quercetin significantly decreased the content of TG (*P* < 0.01), TC (*P* < 0.01), and LDL (*P* < 0.05) in serum. Contents of serum leptin and adiponectin were significantly increased by 0.04% and 0.06% dietary quercetin supplementation (*P* < 0.01) (Table 4).

### 3.3. Summary of the Raw Sequence Reads

Sequence data from the 12 samples were mapped to the reference genome (*Gallus gallus*, 5.0).  Table 5 presents a summary of the RNA-seq analysis including the number of mapped reads and detection of corresponding broilers. The RNA-seq libraries of the 12 samples were sequenced on the Illumina HiSeq 2500 platform, generating 6.22 Gb raw paired-end reads. Comparison of clean reads with the reference genome sequence was done using HISAT. The average ratio of each sample reached 79.09%, and the uniform ratio between samples indicates that the data among the samples were comparable. These 436 million (total) clean reads were subjected to further analysis; 29,186,694 (79.96%), 28,367,936 (78.96%), 29,334,130 (79.55%), and 29,185,088 (80.26%) reads for the control, 0.02%, 0.04%, and 0.06% quercetin groups, respectively, were mapped to the chicken reference genome. The average mapping frequency was 79.68% alignment to the chicken reference genome. On average, 62.27% of the reads were uniquely mapped to the galGal5 (Gallus_gallus-5.0) assembly of the chicken genome, and the mapping frequencies were 62.19%, 61.35%, 62.49%, and 63.05% for the control, 0.02%, 0.04%, and 0.06% quercetin groups, respectively (Table 5). Variant/reference quality ≥ 30 and quality ≥ 20 means that the alternate allele was supported by minimum Phred mass fractions of 30 and 20, and had averages of 98.01% and 93.93%. The above results showed that the next analysis was performed.

### 3.4. Identification of Differentially Expressed Genes

**Figure 1:**
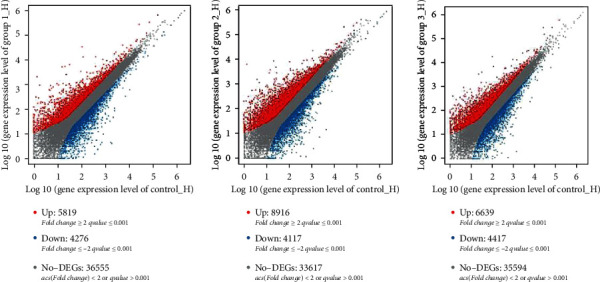
Comparison of differentially expressed genes between control and quercetin (0.02%, 0.04%, and 0.06%). Scatter plot shows the correlation of gene abundance. Red points represent genes upregulated by at least twofold at FDR < 0.05, blue points represent genes downregulated at the same thresholds, and grey dots indicate transcripts that did not change significantly. Group1_H, Group2_H, and Group3_H mean 0.02% quercetin, 0.04% quercetin, and 0.06% quercetin, respectively.

The detection and analysis of DEGs between control and 0.02%, 0.04%, and 0.06% quercetin help elucidate the regulation of genes. Compared with control, 10,095 significant DEGs were found in this study, including 4276 downregulated and 5819 upregulated genes in 0.02% quercetin; 13,033 significant DEGs were found in this study, including 4117 downregulated and 8916 upregulated genes in 0.04% quercetin; and 11,056 significant DEGs were found in this study, including 4417 downregulated and 6639 upregulated genes in 0.06% quercetin (Figure 1).

The top 10 genes were significantly upregulated and downregulated among the samples according to the log_2_^FC^ (Table 6). H3 histone family 3C (H3F3C), histone-lysine N-methyltransferase 2D (KMT2D), PNN-interacting serine- and arginine-rich protein (PNISR), TNF receptor superfamily member 11a (TNFRSF11A), and LOC107052719 were significantly downregulated in 0.02% quercetin/control, 0.04% quercetin/control, and 0.06% quercetin/control. Isocitrate dehydrogenase (NADP (+)) 1 (IDH1), CD36, SON DNA-binding protein (SON), prosaposin (PSAP), and nuclear receptor corepressor 1 (NCOR1) were significantly upregulated in 0.02% quercetin/control, 0.04% quercetin/control, and 0.06% quercetin/control.

### 3.5. Functional Analysis of Differentially Expressed Genes

**Figure 2:**
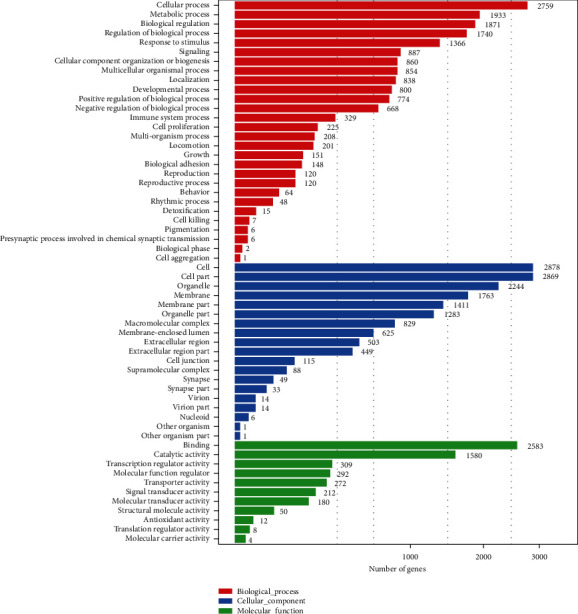
GO analyses of differentially expressed genes in control and quercetin (0.02%, 0.04%, and 0.06%).

The functional distribution of the DEGs was analyzed in the ileum of broilers supplemented with dietary quercetin (0.02%, 0.04%, and 0.06%) compared with the control using GO enrichment and KEGG pathway analyses to understand the regulatory network of lipid metabolism. GO is a type of biological ontology language, which is divided into three parts, including biological process, cellular component, and molecular function. Unlike the functional annotation of a single gene, a gene functional enrichment analysis is based on GO entries, and the results may directly reveal the overall functional characteristics of an entire list of genes. Metabolic processes accounted for the second in the most significantly enriched GO term (Figure 2).

The results showed that 9039 DEGs of control/0.02% quercetin were annotated into 336 pathways, 11842 DEGs of control/0.04% quercetin were annotated into 337 pathways, and 9970 DEGs of control/0.06% quercetin were annotated into 337 pathways, including three different classifications (Table 6). The KEGG database revealed that genes were enriched in the transcriptome participating in 33 pathways related to lipid metabolism.

Compared with control, there were differential gene enrichment pathways including glycerophospholipid metabolism (*P* < 0.01), AMPK signaling pathway (*P* < 0.01), cholesterol metabolism (*P* < 0.01), steroid hormone biosynthesis (*P* < 0.01), insulin resistance (*P* < 0.05), bile secretion (*P* < 0.01), and metabolic pathway (*P* < 0.05) in 0.02% quercetin. There were differential gene enrichment pathways including cholesterol metabolism (*P* < 0.01), insulin resistance (*P* < 0.05), and fatty acid metabolism (*P* < 0.05) in 0.04% quercetin. And there were differential gene enrichment pathways including fat digestion and absorption (*P* < 0.01), glycerophospholipid metabolism (*P* < 0.01), fatty acid degradation (*P* < 0.05), metabolic pathways (*P* < 0.05), and ether lipid metabolism (*P* < 0.05) in 0.06% quercetin (Table 7). The main DEGs in significant signaling pathways were as follows: fat digestion and absorption which included microsomal triglyceride transfer protein (MTTP), apolipoprotein A1 (APO A1), secreted phospholipase A2 (SPLA2) G12B (PLA2G12B), and ATP-binding cassette (ABC) transporter (ABCG5/8); glycerophospholipid metabolism which included selenoprotein I (SELEDI), phospholipase D1 (PLD1), and lysophosphatidylcholine acyltransferase 3 (LPCAT3); AMPK signaling pathway which included CD36, STE20-related kinase adaptor alpha (STRADA), protein kinase AMP-activated noncatalytic subunit beta 2 (PRKAB2), TSC2, and phosphatidylinositol 3-kinase 1 (PI3KR1); cholesterol metabolism which included apolipoprotein A4 (APO A4), apolipoprotein C3 (APO C3), and scavenger receptor class B member 1 (SCARB1); fatty acid metabolism which included acyl-CoA synthetase long-chain family member 4 (ACSL4), acyl-CoA synthetase long-chain family member 3 (ACSL3), acyl-CoA synthetase long-chain family member 5 (ACSL5), alcohol dehydrogenase 1C (ADH1C), and carnitine palmitoyltransferase 1A (CPT1A); and steroid hormone biosynthesis which included cytochrome P450 family 3 subfamily A member 5 (CYP3A5), LOC107080643, and steroid sulfatase (STS) (Table 8).

**Figure 3:**
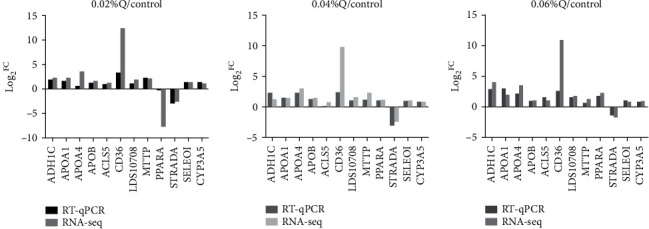
Correlations of mRNA expression level of 12 random DEGs between high and low polyunsaturated fatty acid percentages using RNA-seq and RT-qPCR. *Note*. The *x*- and *y*-axes correspond to the log_2_^(ratio of quercetin/control)^ measured by RNA-seq and RT-qPCR, respectively. Values are mean ± SEM (*n* = 6). 0.02%Q, 0.04%Q, and 0.06%Q mean 0.02% quercetin, 0.04% quercetin, and 0.06% quercetin, respectively.

**Figure 4:**
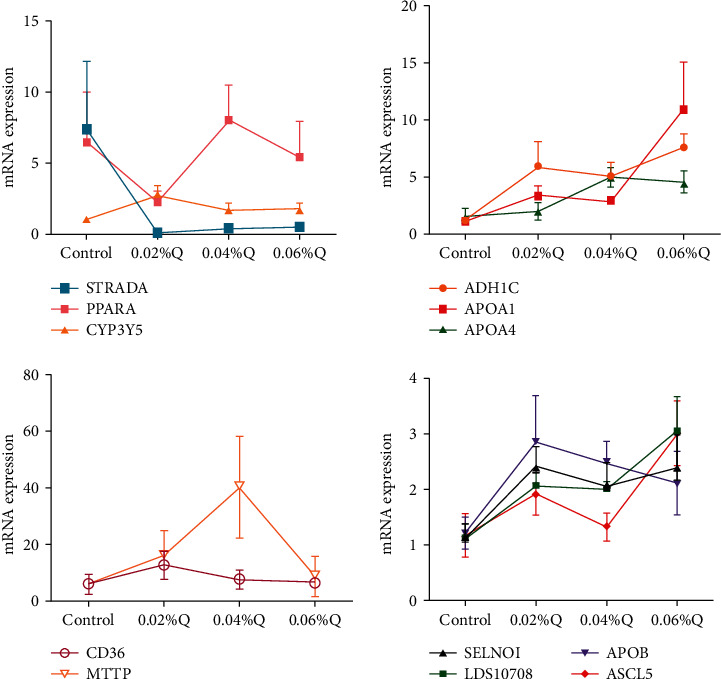
Effects of quercetin on content and mRNA expression of enzymes associated with lipid metabolism in ileal mucosa. *Note*. (1) The results of relative quantification are expressed as 2^−ΔΔCT^. The quantification of control is 1, namely 2^−ΔΔCT^ = 1. The value 2^−ΔΔCT^ of the treatment group is a multiple of control. *n* = 6. (2) Mean values without a common letter are significantly different, *P* < 0.05. Values are mean ± SEM (*n* = 6). 0.02%Q, 0.04%Q, and 0.06%Q mean 0.02% quercetin, 0.04% quercetin, and 0.06% quercetin, respectively.

To verify the accuracy of the RNA-seq results in the transcriptome, the 12 genes from the main lipid metabolic pathways were randomly selected, including 11 significantly upregulated genes and 1 significantly downregulated gene. The expression levels of these 12 genes were quantified using RT-qPCR, and *β*-actin was used as the internal reference gene. The results showed that the vast majority of genes selected by RT-qPCR validation were similar to sequencing data in the expression pattern, and it suggested that the detection and expression abundance of genes in the present transcriptome sequencing was highly accurate (Figures 3 and 4).

## 4. Discussion

### 4.1. The Effect of Quercetin on Carcass Characteristics in Broilers

Flavonoids belong to a group of natural substances, which have a variable phenolic structure and are abundant in fruit, vegetables, tea, and wine. Quercetin is one of the most abundant flavonoids [21]. Different from mammals, the chickens synthesize fatty acids predominantly in the liver and then export fatty acids to other tissues including muscle and adipose tissue by the peripheral vascular system. Therefore, the blood lipid index is related to the carcass characteristics. However, reports concerning the effects of flavonoids on carcass characteristics of animals were also not consistent. 0.20% flavones of sea buckthorn significantly increased dressing percentage [22]. However, the results of the present study showed that no significant responses were found in breast and thigh muscle percentage in quercetin treatments. There was a trend to increase thigh muscle percentage by dietary quercetin supplementation (*P* = 0.078), affirming the findings of Ma et al. [23]. Too much abdominal fat may adversely affect feed conversion rate and the commercial value of the carcass [24]. Diet supplemented with fermented *Ginkgo biloba* leaves (including abundant flavonoids) in broilers decreased abdominal fat deposition [25]. Moreover, abdominal fat deposition was reduced in chickens that consumed Hawthorn extract in drinking water [26]. Our results showed that abdominal fat percentage was lowered by 0.06% quercetin, compared with the other groups (*P* < 0.05) in broilers. The current results are supported by previous studies on the effect of flavonoids on chicken quality in broilers [27, 28].

### 4.2. Effect of Quercetin on Serum Biochemical Parameters in Broilers

Lipids mainly include triglyceride (TG), phospholipids, and cholesterol (CHO), and the content of TG and CHO are key indicators of lipid metabolism. Hesperidin and naringin mainly regulated lipid metabolism by reducing the content of TG and CHO in the human liver [29]. Furthermore, Hawthorn leaf flavonoid (HLF) extract significantly reduced serum TG and TC levels in mice [30]. In addition, naringenin supplementation significantly decreased concentrations of LDL in ethanol-fed rats [31]. *Chrysanthemum indicum* ethyl acetate (CIEA) significantly decreased serum lipid profiles, including TG and LDL [29]. The current results showed that 0.04% and 0.06% quercetin significantly decreased the content of serum TG, TC (*P* < 0.01), and LDL (*P* < 0.05) in broilers. This further confirmed the hypolipidemic effects of quercetin, just like the other flavonoids.

Leptin is a hormone synthesized by white adipose tissue (WAT) and plays an important role in weight control by suppressing food intake and increasing energy expenditure. Leptin is also a regulator of cellular TG content [32]. Adiponectin is involved in fatty acid oxidation and glucose regulation in liver [33, 34]. And, adiponectin decreased TG content in the muscles and livers of obese mice [35]. CIEA increased adiponectin levels in HFD-induced obese mice [29]. Moreover, the level of leptin (556.7 pg/ml) was significantly increased in mice that received 150 mg/kg *M. citrifolia* leaf extracts (MLE60) compared to control (535.3 pg/ml) [36]. The result of adiponectin was consistent with the study on *Poncritus trifoliate* leaf extracts which increased adiponectin levels in serum of HFD-fed mice [37]. The present results showed that 0.04% and 0.06% quercetin significantly increased serum adiponectin and leptin levels (*P* < 0.01). Meanwhile, together with the results for TG in our experiment, our findings were supported by Nepali et al. and Ma et al. [23, 29], who found that leptin and adiponectin directly interacted with TG, thus decreasing abdominal fat percentage.

### 4.3. The Effect of Quercetin on Lipid Metabolism Mechanism in Ileum of Broilers

Recently, the RNA-seq technique has been a powerful and revolutionary approach to quantify gene expression levels and survey detailed transcriptome profiles at unprecedented resolution and sensitivity [38, 39]. In chickens, the intestinal mucosa [17], heart [18], and uterine [19] and ovarian tissues [20] have been determined using RNA-seq. Some studies found that quercetin regulated lipid metabolism [30, 31]. Nonetheless, the precise regulating mechanism is not clear in the ileum of broilers. In the present study, RNA-seq was used for determining gene expression profiles and metabolic pathways in broilers fed by diet supplemented with dietary quercetin. Meanwhile, multiple signaling pathways were detected, including the metabolism process in the ileum of broilers. The current RNA-seq data provided greater sequence depth and obtained 79.09% proportions of mapped reads. The high-quality sequences and superior mapping rates enabled the accuracy and reliability of further differential gene expression (Table 4).

In the present study, among the top 10 of the significantly upregulated and downregulated genes between quercetin (0.02%, 0.04%, and 0.06%) and the control group according to the log_2_^FC^, the upregulated genes are mainly related to amino acid metabolism (H3F3C, KMT2D, and PNISR), while the downregulated genes are mainly related to lipid metabolism (IDH1, PSAP, and CD36). IDH1 is involved in the tricarboxylate cycle and plays a role in energy metabolism and biosynthesis in organisms. PSAP is a lysosomal gene which delivers bound sphingolipids to cell plasma membranes and enters an endocytotic pathway, responsible for digesting the lipoproteins [39–41]. CD36 is a membrane protein associated with fatty acid transportation [42].

After functional enrichment analyses, GO terms and KEGG pathways were mainly involved in cellular and metabolism processes (Figure 3). The results were also in accordance with the previous studies in chickens [43]. These results suggested that lipid metabolism mainly proceeded in the intestine. In the meantime, to confirm the putative results from RNA-seq, several genes of the lipid metabolic pathway for RT-qPCR assays were randomly selected. Overall, there was excellent agreement and high concordance between the computational and experimental results, which were similar to some previous results in cows [44], broilers [45], and chickens [46]. In the present study, thirty-three of the significant lipid metabolic pathways were detected compared to the control, and most of them were involved in fat digestion and absorption, glycerophospholipid metabolism, AMPK signaling pathway, fatty acid degradation, and cholesterol metabolism (Table 6).

#### 4.3.1. Fat Digestion and Absorption

There were 115 DEGs in this pathway, and 0.06% quercetin was significantly different from the control (*P* < 0.01). APO A1, MTTP, ABCG5/8, and PLA2G12B are important genes involved in fat digestion and absorption, which are highly expressed in 0.02%, 0.04%, and 0.06% quercetin treatments. Secreted PLA2G12B is a novel mediator of TG metabolism [47]. Intriguingly, the plasma TG, TC, and fatty acid levels were apparently decreased in PLA2G12B-null mice, which are attributed to the compromised hepatic VLDL-TG secretion. ABCG5/ABCG8 belongs to the ABC transporter family members and mediates the biliary secretion of cholesterol. Polydatin increased the secretion of cholesterol into bile by ABCG5/ABCG8, thus improving cholesterol metabolism [48]. The protein expression of MTTP was significantly increased by fisetin [49]. MTTP mainly acts on the synthesis and secretion of chylomicrons and VLDL in the lumen of the endoplasmic reticulum of the liver and the intestine [50, 51]. Numerous studies proved that MTTP plays a distinct role in lipid transport [52, 53]. MTTP is involved in delivering TG to nascent APO B molecules during the assembly of lipoprotein particles [54]. Our results showed that 0.02%, 0.04%, and 0.06% quercetin upregulated MTTP with a log_2_^FC^ of 2.01, 2.16, and 1.61, respectively. Furthermore, another significantly differential gene, APO A1, belongs to an APO family that encodes important regulators of lipid biosynthesis and metabolism [55]. APO A1 is involved in cholesterol transport [54] and the major constituent of the protein fraction of HDL. Quercetin increased APO A1 mRNA and gene promoter activity in HepG2 cells [56]. According to our log_2_^FC^ records, the expression of APO A1 was increased by 2.1-, 1.37-, and 1.95-fold as much as the control for 0.02%, 0.04%, and 0.06% quercetin. Meanwhile, together with the results for HDL in this experiment, our findings were supported by Zhou et al. [57], who found that APO A1 directly interacted with HDL. The above results indicate that quercetin regulated lipid metabolism through fat digestion and the absorption pathway of upregulating APO A1, MTTP, ABCG5/8, and PLA2G12B.

#### 4.3.2. Glycerophospholipid Metabolism

PLD1 and LPCAT3 are the two main genes that are associated with the glycerophospholipid metabolism. There were 105 DEGs in this pathway, and 0.02% quercetin and 0.06% quercetin were significantly different from control (*P* < 0.01). There are four isoforms for LPCAT (LPCAT1, LPCAT2, LPCAT3, and LPCAT4) [58]. Plasma VLDL-TG levels were reduced in the liver of mice lacking LPCAT3 [59]. LPCAT3 deficiency significantly reduced polyunsaturated phosphatidylcholines (PCs) on the hepatocytes and enterocytes, and impacted plasma lipid metabolism [60]. Again, LPCAT3 activity was an important determinant of SREBP-1c activation and lipogenesis [61]. PLD hydrolyzes phosphatidylcholine producing phosphatidic acid, and there are two main isoforms: PLD1 and PLD2. PLD1, the key enzyme involved in lipid metabolism, is associated with metastasis [62]. PLD1 knockout mice resulted in mitochondrial abnormalities and subsequent lipid accumulation [63]. PLD1 knockout mice consume more food due to defects in the hypothalamus, which results in obesity [64]. Furthermore, hepatic TG and TC were increased by 26.5% and 60.4%, respectively, in 4-week high-fat-diet- (HFD-) fed PLD1 knockout mice [63]. The above results indicate that quercetin regulated lipid metabolism through the glycerophospholipid metabolism pathway of upregulating LPCAT3 and PLD1.

#### 4.3.3. AMP-Activated Protein Kinase (AMPK) Signaling Pathway

There were 505 DEGs in this pathway, and 0.02% quercetin was significantly different from control (*P* < 0.01). The CD36, PRKAB2, and PI3KR1 genes involved in the AMPK signaling pathway were differentially expressed compared with the control. CD36 is a membrane receptor that facilitates long-chain fatty acid uptake and plays an important role in mitochondrial oxidation [65, 66]. A series of evidence supports that CD36 may promote the clearance of chylomicrons from plasma [67, 68] as well as the lipid metabolism and fatty acid transport [65, 66, 69, 70]. CD36 enhanced cellular fatty acid (FA) uptake using the overexpressing CHO model [71]. Our results showed that 0.02%, 0.04%, and 0.06% quercetin increased CD36 expression, which was consistent with Cabanillas et al.'s findings [72]. PI3KR encodes p58 [73]; meanwhile, p58 plays a negative role in PI3K/AKT signaling axis [74, 75]. The PI3K/AKT signaling pathway regulates lipid metabolism. Therefore, quercetin downregulated the PI3KR1 gene that activated the PI3K/AKT signaling axis, thus regulating lipid metabolism. However, PRKAB2 encodes AMPK, which is an energy sensing/signaling intracellular protein activated by an increase in the cellular AMP : ATP ratio after ATP depletion. Once activated, AMPK inhibits fatty acid synthesis [76]. Quercetin regulated lipid metabolism by the AMPK signaling pathway in the liver (data not published). In addition, apigenin suppresses adipogenesis in 3T3-L1 cells via activation of the AMPK pathway [77]. Our results are in line with previous studies, in which quercetin upregulated the AMPK signaling pathway [78].

#### 4.3.4. Fatty Acid Degradation

In the present study, compared with the control, 0.06% quercetin was significantly different from the control in the fatty acid degradation pathway (*P* < 0.05). ACSLs (ACSL3, ACSL4, and ACSL5), ADH1C, and CPT1A were upregulated by dietary quercetin supplementation. ACSLs are essential for de novo lipid synthesis, fatty acid catabolism, and remodeling of membranes [79]. A previous study identified the sequences of the genes of the ACSL family (ACSL1, ACSL3, ACSL4, ACSL5, and ACSL6) in the sable [80] via transcriptome sequencing. ACSL3 mediated hepatic lipogenesis through transcriptional regulation of lipogenic gene expression [81]. It was transcriptionally upregulated by the cytokine oncostatin M (OSM) in HepG2 cells, accompanied by reduced cellular TG content and enhanced *β*-oxidation [82]. Polymorphisms of the ACSL4 gene were significantly correlated with liver and intramuscular fat content [83, 84]. Recently, the first evidence in vivo showed that ACSL4 plays a role in plasma TG and hepatic phospholipid synthesis of hyperlipidemic mice [85]. Meanwhile, ACSL5 is an important regulator of whole-body energy metabolism and is implicated in TG synthesis and fat deposition [86–88]. Knockdown of ACSL5 in isolated rat hepatocytes reduces TG accumulation and increases fat oxidation [89]. Our results further confirmed that 0.06% quercetin significantly decreased the plasma's TG content via increasing expression of ACSL3, ACSL4, and ACSL5 (*P* < 0.05). ADH1C (also known as ADH3) is the predominant isozyme expressed in duodenal, jejunal, and ileum mucosa [90], which has two alleles, namely gamma 1 and gamma 2 (*γ*1 and *γ*2). It also directly interacted with the high-density lipoprotein (HDL) [91]. The present study showed that 0.06% quercetin significantly increased mRNA expression of ADH1C in broilers (*P* < 0.01), while ACSLs and ADH1C directly interacted with TG and HDL, respectively, together with the results on TG and HDL in this experiment, thereby decreasing abdominal fat percentage.

#### 4.3.5. Cholesterol Metabolism

APO A4, APO B, APO C3, and SCARB1 are key transcription factors in the regulation of cholesterol metabolism. There were 217 DEGs in this pathway, and 0.02% quercetin and 0.04% quercetin were significantly different from the control (*P* < 0.01). APO A4, APO B, and APO C3 also belong to an APO family that encodes important regulators of lipid biosynthesis and metabolism [54]. APO A4 is involved in TG metabolism [59]. Additionally, APO A4 knockout mice as a regulator of TG metabolism increased plasma TG [92]. The results of the current study showed that 0.04% quercetin significantly increased the expression of APO A4 in the ileum of broilers (*P* < 0.05). APO B located on the surface of the lipoprotein particles is the main lipoprotein of low-density lipoprotein taking charge of transferring cholesterol into tissues. Total flavonoids extracted from *Polygonum perfoliatum* L. (TFP) decreased the level of APO B and increased the level of APO A, thus adjusting the ratio of APO A/APO B and the metabolic disturbance of lipoprotein by TFP treatment in hyperlipidemia rats [93]. However, our results showed that 0.02%, 0.04%, and 0.06% quercetin increased expression of APO B in the ileum of broilers. Since ileum mucosa has several characteristics different from the liver, the regulatory mechanisms of the respective genes possibly differ [94]. APO C3 is an exchangeable lipoprotein produced by both the liver and the intestine, and found on both chylomicrons and VLDLs. An increase in intestinal APO C3 expression may be of clinical importance for the control of hypertriglyceridemia and the metabolic syndrome [95]. Meanwhile, studies suggested that HDL stimulates scavenger receptor class B type 1 (SR-B1) to promote hepatic uptake of cholesterol. SR-B1 is encoded by the SCARB1 gene in humans, which has been linked to cholesterol metabolism in humans [96]. Our results showed that quercetin decreased TG, TC, and LDL via upregulating APO A4, APO B, APO C3, and SCARB1 expression.

In summary, our results showed that leptin and adiponectin directly interacted with TG, thus decreasing abdominal fat percentage. Meanwhile, quercetin decreased TG, TC, and LDL via regulating APO A4, APO B, APO C3, APO A1, MTTP, ABCG5/8, PLA2G12B, CD36, PRKAB2, PIK3R1, ACSLs, ADH1C, LPCAT3, PLD1, and SCARB1 gene expression in ileal mucosa of broilers.

## 5. Conclusions

Quercetin decreased abdominal fat percentage through regulating the signaling pathway, including fat digestion and absorption, glycerophospholipid metabolism, the AMPK signaling pathway, fatty acid degradation, and cholesterol metabolism.

## Figures and Tables

**Table 1:** Analysis composition of basal diets and nutrient level (air-dry basis, %).

Item	Content (1 to 3 week)	Content (4 to 6 week)
Ingredient		
Corn	57.50	62.30
Soybean meal	34.50	30.00
Vegetable oil	3.00	3.00
Fish meal	1.00	1.00
Methionine	0.20	0.20
Dicalcium phosphate	1.62	1.67
Limestone	1.55	1.20
Sodium chloride	0.30	0.30
Multivitamin premix^1^	0.03	0.03
Mineral premix^1^	0.20	0.20
Choline	0.10	0.10
Total	100.00	100.00
Nutrient^2^		
Metabolizable energy (ME) (MJ/kg)	12.33	12.50
CP	21.75	19.72
Total lysine (%)	1.18	1.04
Methionine (%)	0.91	0.86
Ca	1.07	0.96
Total P	0.70	0.68
Available P	0.46	0.45

^1^Amount provided per kilogram of diet: vitamin A = 1,500 IU; vitamin D_3_ = 3,200 IU; vitamin E = 10 IU; vitamin K = 0.5 mg; vitamin B_1_ = 1.8 mg; vitamin B_2_ = 3.6 mg; vitamin B_6_ = 3.5 mg; vitamin B_12_ = 0.01 mg; biotin = 0.15 mg; folic acid = 0.55 mg; niacin = 30 mg; pantothenic acid = 10 mg; Cu (CuSO_4_·5H_2_O) = 8 mg; I (KI) = 0.35 mg; Fe (FeSO_4_·7H_2_O) = 80 mg; Mn (MnSO_4_·H_2_O) = 60 mg; Se (NaSeO_3_) = 0.15 mg; Zn (ZnO) = 40 mg. ^2^Based on composition of ingredients provided by NY/T33-2004.

**Table 2:** Parameters of primer pairs for the genes.

Gene	Primer sequence	GenBank accession
MTTP	F: 5′-GCTGGTAATGCTGAGGTGGATTCC-3′	NM_001109784.2
	R: 5′-AGGAGAGCCAACTCTGTCCATCTG-3′	

APOA1	F: 5′-CCTTCTGGCAGCACGATGAGC-3′	XM_015297971.2
	R: 5′-CAGCGTGTCCAGGTTGTCAGC-3′	

CD36	F: 5′-ACCAGACCAGTAAGACCGTGAAGG-3′	NM_001030731.1
	R: 5′-ATGTCTAGGACTCCAGCCAGTGTG-3′	

STRADA	F: 5′-ACACCACCACAATTCCTGCTGATG-3′	XM_025143971.1
	R: 5′-TGACTCTCCGTTGGCTGCTCTC-3′	

APOA4	F: 5′-GACAACGCCGACAGCATCCAG-3′	NM_204938.2
	R: 5′-TCCACGCTCTGTGCCACCTG-3′	
APOB	F: 5′-AGGTGGTGGTGAAGAGGTGGAGAG-3′	NM_001044633.1
	R: 5′-GAGCAGCAAGAGCCGCACAG-3′	

CYP3A5	F: 5′-AGCCTGCGGTTGTTGTCATGG-3′	NM_001001751.2
	R: 5′-CTGCGGTTGGTGAAGGTGGAG-3′	

LOC107080643	F: 5′-ATCCTCTGCGTCGCTCTCCATC-3′	NM_001318851.1
	R: 5′-TACACTCGCGTCCACCGTCAG-3′	

ACSL5	F: 5′-GGTTCACAAGGAGAGTGCAGGAAG-3′	NM_001031237.1
	R: 5′-TCTGAGGCTAGGAGCAGGAAGTTC-3′	

ADH1C	F: 5′-TTGCCACAACTACGGAGTCAG-3′	NM_001305183.1
	R: 5′-CCAGGTGCGACCACTGAAGATAAG-3′	

SELENOI	F: 5′-GCCTCTGAACTGGATGCTGCTG-3′	NM_001031528.3
	R: 5′-TGGCTCACCACGACCACTCC-3′	

PPAR*α*	F: 5′-TGCTGTGGAGATCGTCCTGGTC-3′	XM_015289959.2
	R: 5′-CTGTGACAAGTTGCCGGAGGTC-3′	

*β*-Actin	F: 5′-TGCGTGACATCAAGGAGAAG-3′	L08165
	R: 5′-TGCCAGGGTACATTGTGGTA-3′	

18sRNA	F: 5′-TAGATAACCTCGAGCCGATCGCA-3′	AF 173612
	R: 5′-GACTTGCCCTCCAATGGATCC TC-3′	

**Table 3:** Effect of quercetin on carcass characteristic in broilers.

Items	Control	0.02% quercetin	0.04% quercetin	0.06% quercetin	*P*
Breast muscle (%)	29.65 ± 0.84^A^	27.11 ± 0.51^B^	29.28 ± 0.57^AB^	30.58 ± 0.4^A^	0.001
Thigh muscle (%)	19.56 ± 0.5	21.58 ± 0.43	20.57 ± 0.6	21.00 ± 0.63	0.078
Abdominal fat (%)	1.68 ± 0.08^A^	1.65 ± 0.07^A^	1.49 ± 0.04^AB^	1.36 ± 0.06^B^	0.004

Note: in the same row, values with different small letter superscripts mean significant difference (*P* < 0.05); values with different capital letter superscripts mean significant difference (*P* < 0.01); Values with no letter or the same letter superscripts mean no significant difference (*P* < 0.05). Values are expressed as mean ± SEM, and *n* = 6 for all groups.

**Table 4:** Effect of quercetin on serum biochemical parameters in broilers.

Items	Control	0.02% quercetin	0.04% quercetin	0.06% quercetin	*P*
TG (mg/dl)	0.68 ± 0.03^A^	0.60 ± 0.03^AB^	0.54 ± 0.03^BC^	0.50 ± 0.03^C^	0.003
LDL (mg/dl)	14.5 ± 1.03^a^	12.4 ± 1.39^ab^	9.65 ± 1.05^b^	9.56 ± 0.99^b^	0.014
HDL (mg/dl)	1.76 ± 0.16	1.69 ± 0.18	2.08 ± 0.16	1.99 ± 0.15	0.298
TC (mg/dl)	4.51 ± 0.19^A^	3.76 ± 0.31^AB^	3.33 ± 0.25^B^	3.36 ± 0.73^B^	0.008
Leptin (ng/ml)	12.47 ± 0.20^A^	10.47 ± 0.19^B^	14.23 ± 0.26^aC^	15.01 ± 0.20^bC^	0.000
Adiponectin (mg/ml)	95.44 ± 4.37^A^	94.55 ± 1.68^A^	109.48 ± 4.47^B^	111.02 ± 3.99^B^	0.004

Note: in the same row, values with different small letter superscripts mean significant difference (*P* < 0.05); Values with different capital letter superscripts mean significant difference (*P* < 0.01); Values with no letter or the same letter superscripts mean no significant difference (*P* > 0.05). Values are expressed as mean ± SEM, and *n* = 6 for all groups.

**Table 5:** Summary statistics for sequence quality and alignment information of 12 samples from ileal mucosa.

Groups	Control	0.02% quercetin	0.04% quercetin	0.06% quercetin
Clean reads	36,504,566	35,926,566	36,864,268	36,359,240
Q20 (%)	97.94	97.97	98.12	98.00
Q30 (%)	93.85	93.89	94.03	93.95
Total mapped reads	29,186,694	28,367,936	29,334,130	29,185,088
Uniquely mapped reads	22,698,908	22,040,620	23,043,483	22,925,773
Multiple mapped reads	6,487,786	6,327,316	6,290,646	6,259,315
Total mapping ratio (%)	79.96	78.96	79.55	80.26
Uniquely mapping ratio (%)	62.19	61.35	62.49	63.05

^1^Uniquely mapped reads = reads that matched only one position in the genome. ^2^Mapping ratio = mapped reads/clean reads. ^3^Unique mapping ratio = mapped unique reads/clean reads. ^4^*n* = 3 for all groups.

**Table 6:** The upregulated and downregulated genes compared with control.

Gene ID	Gene name	Log_2_^FC^	*q* value	*P* value	Type
Downregulated genes					
0.02% quercetin/control					
NM_001031482.2	H3F3C	-10.898	3.40*E* − 138	2.41*E* − 139	H3F3C: H3 histone, family 3C
XM_025145484.1	KMT2D	-10.7913	1.16*E* − 130	8.94*E* − 132	KMT2D: histone-lysine methyltransferase 2D
XM_025148752.1	PNISR	-10.5709	2.55*E* − 116	2.28*E* − 117	PNISR: PNN-interacting serine- and arginine-rich protein
XM_004939689.3	TNFRSF11A	-10.5566	1.92*E* − 115	1.73*E* − 116	TNFRSF11A: TNF receptor superfamily member 11a
0.04% quercetin/control					
XM_015282677.2	LOC107052719	-13.92476	0	0	Uncharacterized LOC107052719
NM_001031482.2	H3F3C	-10.9586	5.51*E* − 140	7.24*E* − 141	H3 histone, family 3C
XM_025145484.1	KMT2D	-10.8519	2.22*E* − 132	3.15*E* − 133	Lysine methyltransferase 2D
XM_025148752.1	PNISR	-10.6903	7.98*E* − 117	1.34*E* − 117	PNN-interacting serine- and arginine-rich protein
XM_004939689.3	TNFRSF11A	-10.6172	5.08*E* − 117	8.47*E* − 118	TNF receptor superfamily member 11a
0.06% quercetin/control					
XM_015282677.2	LOC107052719	-13.9034	0	0	Uncharacterized LOC107052719
NM_001031482.2	H3F3C	-10.9373	3.19*E* − 139	2.53*E* − 140	H3 histone, family 3C
Upregulated genes					
0.02% quercetin/control					
XM_015289550.2	IDH1	13.64523	5.78*E* − 153	3.61*E* − 154	Isocitrate dehydrogenase (NADP (+)) 1, cytosolic
XM_025147445.1	CD36	12.3756	2.82*E* − 288	8.11*E* − 290	CD36 molecule
XM_003640519.4	SON	13.03347	0	0	SON DNA-binding protein
XM_015288195.2	PSAP	11.55249	2.69*E* − 189	1.34*E* − 190	Prosaposin
XM_004946661.2	NCOR1	11.06195	1.02*E* − 146	6.73*E* − 148	Nuclear receptor corepressor 1
0.04% quercetin/control					
XM_015288195.2	IDH1	12.66957	7.90*E* − 97	1.61*E* − 97	Isocitrate dehydrogenase (NADP (+)) 1, cytosolic
XM_015288195.2	PSAP	13.97177	0	0	Prosaposin
XM_003640519.4	SON	12.38731	2.36*E* − 296	1.18*E* − 297	SON DNA-binding protein
0.06% quercetin/control					
XM_025147445.1	CD36	10.91717	6.73*E* − 138	5.43*E* − 139	CD36 molecule
XM_004946661.2	NCOR1	10.70961	1.18*E* − 123	1.11*E* − 124	Nuclear receptor corepressor 1
XM_004946661.2	NCOR1	11.06195	1.02*E* − 146	6.73*E* − 148	Nuclear receptor corepressor 1

^1^ *q* value: the corrected *P* value. The smaller the *q* value, the more significant the difference in gene expression. ^2^*P* value: significant statistical value. ^3^Log_2_^fold change^(sample 2/sample 1): differential expression multiple between samples (groups) after log_2_ conversion.

**Table 7:** Important lipid metabolic pathways.

Pathway ID	Pathway definition	*P* value			All genes
		Control/0.02% quercetin	Control/0.04% quercetin	Control/0.06% quercetin	
Ko04975	Fat digestion and absorption	8.137809*E* − 05	0.05434665	0.004701606	115
Ko00564	Glycerophospholipid metabolism	0.01281478	0.0535793	0.009868102	105
Ko04152	AMPK signaling pathway	0.001574863	1.896442*E* − 07	0.09219744	505
Ko04979	Cholesterol metabolism	0.001932857	0.008406933	0.08742154	217
Ko00071	Fatty acid degradation	0.06928025	0.06733572	0.03222657	117
Ko00140	Steroid hormone biosynthesis	0.003068663	0.09103691	0.1157436	188
Ko04931	Insulin resistance	0.0307667	0.03297987	0.5826344	477
Ko00120	Primary bile acid biosynthesis	0.3000608	0.9761336	0.1666659	42
Ko00561	Glycerolipid metabolism	0.22205	0.4289608	0.3153083	457
Ko04910	Insulin signaling pathway	0.2856451	0.541523	0.6822437	702
Ko04923	Regulation of lipolysis in adipocytes	0.3564451	0.1735033	0.7989723	177
Ko00591	Linoleic acid metabolism	0.3872519	0.746023	0.4859596	112
Ko00592	Alpha-linolenic acid metabolism	0.4011347	0.620726	0.28578	96
Ko00590	Arachidonic acid metabolism	0.4455902	0.2346687	0.1989776	178
Ko03320	PPAR signaling pathway	0.5193936	0.719966	0.9634836	501
Ko00061	Fatty acid biosynthesis	0.6108374	0.04648987	0.5227091	72
Ko00073	Cutin, suberine, and wax biosynthesis	0.6533068	0.1904358	0.7331227	25
Ko00785	Lipoic acid metabolism	0.6816958		0.4859596	6
Ko01040	Biosynthesis of unsaturated fatty acids	0.7195848	0.3515127	0.759634	84
Ko00072	Synthesis and degradation of ketone bodies	0.7962817	0.2089213	0.2889255	57
Ko00062	Fatty acid elongation	0.798622	0.1710817	0.9398205	83
Ko04151	PI3K/AKT signaling pathway	0.9998879	0.6963632	0.8758061	1862
Ko04024	cAMP signaling pathway	0.9999875	0.9999477	0.9999958	993
Ko04920	Adipocytokine signaling pathway	0.70824614	0.07274647	0.2555452	278
Ko00565	Ether lipid metabolism	0.09687652	0.08454478	0.04087001	183
Ko01100	Metabolic pathways	0.02313553	0.1280044	0.044613536	5281
Ko00062	Fatty acid elongation	0.798622	0.1710817	0.9398205	83
Ko00603	Glycosphingolipid biosynthesis—globo and isoglobo series	0.1741715	0.1247949	0.4047945	506
Ko04911	Insulin secretion	0.999995	1	0.9999993	549
Ko00600	Sphingolipid metabolism	0.4227905	0.1776699	0.5005832	165
Ko00604	Glycosphingolipid biosynthesis—ganglio series	0.4087944	0.1547651	0.449979	74
Ko04976	Bile secretion	0.001000504	0.3618376	0.4639476	230
Ko01212	Fatty acid metabolism	0.45523	0.0157721	0.4646036	173

^1^ *P* value: significant statistical value.

**Table 8:** Summary of DEGs involved in lipid accumulation.

Gene ID	Gene name	Log_2_^FC^			Type
		0.02% quercetin/control	0.04% quercetin/control	0.06% quercetin/control	
NM_001109784.2	MTTP	2.01	2.16	1.61	Fat digestion and absorption
XM_015297971.2	APOA1	2.1	1.37	1.95	Fat digestion and absorption
XM_421584.6	PLA2G12B	1.63	1.02	1.2	Fat digestion and absorption
XM_419457.6	ABCG5	1.12	0.82	0.8	Fat digestion and absorption
XM_419458.6	ABCG8	1.56	0.53	1.34	Fat digestion and absorption
NM_001031528.3	SELENOI	1.21	0.92	0.73	Glycerophospholipid metabolism
XM_015291712.1	PLD1	6.86	5.66	7.22	Glycerophospholipid metabolism
XM_416516.5	LPCAT3	1.38	1.15	1.34	Glycerophospholipid metabolism
NM_001030731.1	CD36	12.38	9.72	10.92	AMPK signaling pathway
XM_025143971.1	STRADA	-2.41	-2.29	-1.55	AMPK signaling pathway
XM_015290312.2	PRKAB2	8.1	6.67	6.91	AMPK signaling pathway
XM_015294450.2	TSC2	-8.23	-8.29	-8.27	AMPK signaling pathway
XM_004937312.3	PIK3R1	-5.93	-5.99	-5.97	AMPK signaling pathway
NM_204938.2	APOA4	3.42	2.94	3.48	Cholesterol metabolism
NM_001044633.1	APOB	1.53	1.34	0.93	Cholesterol metabolism
NM_001302127.1	APOC3	3.29	3.15	3.55	Cholesterol metabolism
XM_015275626.1	SCARB1	8.06	6.02	6.62	Cholesterol metabolism
NM_001001751.2	CYP3A5	1.02	0.7	0.88	Steroid hormone biosynthesis
NM_001318851.1	LOC107080643	1.71	1.46	1.71	Steroid hormone biosynthesis
XM_025146627.1	STS	3.64	3.64	3.8	Steroid hormone biosynthesis
XM_015278614.2	ACSL4	8.67	9.35	6.45	Fatty acid degradation
XM_0152777033.2	ACSL3	3.59	3.99	2.67	Fatty acid degradation
NM_001031237.1	ACSL5	1.17	0.68	0.96	Fatty acid degradation
NM_001305183.1	ADH1C	2.1	1.2	3.94	Fatty acid degradation
XM_015286797.2	CPT1A	7.63	7.63	8.6	Fatty acid degradation

^1^Log_2_^fold change^(sample 2/sample 1): differential expression multiple between samples (groups) after log_2_ conversion.

## Data Availability

Answer: Yes. Comment:

## Abbreviations

HDL: High-density lipoprotein
TC: Total cholesterol
TG: Triglyceride
LDL: Low-density lipoprotein
RT-qPCR: Real-time quantitative PCR
GO: Gene Ontology
KEGG: Kyoto Encyclopedia of Genes and Genomes
FA: Fatty acid
H3F3C: H3 histone family 3C
KMT2D: Histone-lysine N-methyltransferase 2D
PNISR: PNN-interacting serine- and arginine-rich protein
TNFRSF11A: TNF receptor superfamily member 11a
IDH1: Isocitrate dehydrogenase (NADP (+)) 1
SON: SON DNA-binding protein
PSAP: Prosaposin
NCOR1: Nuclear receptor corepressor 1
MTTP: Microsomal triglyceride transfer protein
APOA1: Apolipoprotein A1
PLA2G12B: Secreted phospholipase A2 (SPLA2) G12B
ABCG5/8: ATP-binding cassette (ABC) transporter 5/8
SELEDI: Glycerophospholipid metabolism included selenoprotein I
PLD1: Phospholipase D1
LPCAT3: Lysophosphatidylcholine acyltransferase 3
STRADA: STE20-related kinase adaptor alpha
PRKAB2: Protein kinase AMP-activated noncatalytic subunit beta 2
PI3KR1: Phosphatidylinositol 3-kinase 1
APO A3/4/5: Apolipoprotein A3/4/5
SCARB1: Scavenger receptor class B member 1
ACSL3/4/5: Acyl-CoA synthetase long-chain family member 3/4/5
ADH1C: Alcohol dehydrogenase 1C
CPT1A: Carnitine palmitoyltransferase 1A
CYP3A5: Cytochrome P450 family 3 subfamily A member 5
STS: Steroid sulfatase
CHO: Cholesterol
HLF: Hawthorn leaf flavonoids
CIEA: *Chrysanthemum indicum* ethyl acetate
WAT: White adipose tissue
AMPK: AMP-activated protein kinase
TFP: Total flavonoids extracted from *Polygonum perfoliatum* L.
SR-B1: Scavenger receptor class B type 1
PCs: Polyunsaturated phosphatidylcholines
HDL: High-density lipoprotein
OSM: Oncostatin M
DEGs: Differentially expressed genes.
